# Gastric Cancer Cell-Derived Exosomal microRNA-23a Promotes Angiogenesis by Targeting PTEN

**DOI:** 10.3389/fonc.2020.00326

**Published:** 2020-03-13

**Authors:** Jiang Du, Yuan Liang, Ji Li, Jin-Ming Zhao, Xu-Yong Lin

**Affiliations:** ^1^Department of Pathology, The First Affiliated Hospital and College of Basic Medical Science, China Medical University, Shenyang, China; ^2^Medical Oncology Department of Thoracic Cancer (2), Cancer Hospital of China Medical University, Liaoning Cancer Hospital & Institute, Shenyang, China

**Keywords:** exosome, gastric cancer, microRNA-23a, PTEN, AKT pathway, angiogenesis

## Abstract

Hypoxia-exposed lung cancer-released exosomal microRNA-23a (miR-23a) has been shown to enhance angiogenesis as well as vascular permeability, contributing to the close correlation between exosomal miR-23a and tumorigenesis. The current study aimed to investigate whether gastric cancer (GC) cell-derived exosomal miR-23a could induce angiogenesis and to elucidate the potential mechanisms associated with the process. Differentially expressed miRNAs in GC were initially screened from the Gene Expression Omnibus database. Target genes were selected following miRNA-mRNA prediction and subsequently verified by dual luciferase reporter assay. RT-qPCR was conducted to detect miR-23a and PTEN expression in GC tissues, cells and exosomes. Human umbilical venous endothelial cells (HUVECs) were co-cultured with GC cell-derived exosomes to assess the angiogenesis mediated by exosomes *in vitro*. Additionally, PTEN was overexpressed in HUVECs to analyze the mechanism by which miR-23a regulates angiogenesis. miR-23a was highly expressed in GC tissues and cells and GC cell-derived exosomes. Angiogenesis was promoted by the co-culture of HUVECs and GC cells-derived exosomes, as evidenced by the increased expression of VEGF but decreased expression of TSP-1. PTEN was targeted by miR-23a and was lowly expressed in GC tissues. In a co-culture system, miR-23a carried by GC cells-derived exosomes promoted angiogenesis via the repression of PTEN. Collectively, GC cell-derived exosomal miR-23a could promote angiogenesis and provide blood supply for growth of GC cells. This study contributes to advancement of miRNA-targeted therapeutics.

## Introduction

Gastric cancer (GC) is a heterogeneous malignancy with epidemiologic and histopathologic differences around the world (1). Global cancer statistics from 2018 revealed that over 1 million people were newly diagnosed with GC resulting in ~0.78 million deaths (2). At present, surgical intervention remains the only curative approach for GC, while adjuvant and neoadjuvant therapy is usually combined with surgery in locally advanced cases (3). Exosomes have emerged as effective drug and gene therapeutic transporters for a wide variety of pathologies (4). Exosomes are cell-derived vesicles with diameter ranging from 20 to 100 nm, capable of influencing angiogenesis, metastasis and other biological cellular properties related to tumorigenesis (5). EGFR delivered by exosomes has been reported to contribute to the liver microenvironment to accelerate liver-specific metastasis of GC (6). Cancer cell-derived exosomes facilitate GC progression by transferring foreign microRNAs (miRNAs), which has shed new light on the mechanism of tumorigenesis and potential novel targets for GC treatment (7). Additionally, genetic and epigenetic abnormalities such as changes in miRNA and gene profiles have been widely implicated in gastric carcinogenesis (8, 9).

miRNAs, a group of small non-coding RNA molecules ~19–25 nucleotides, are dysregulated in GC, and some of them are related with GC progression and prognosis (10). miRNAs are important mediators of gene expression that guide their inhibition via pairing to messenger RNAs (mRNAs) of protein-coding genes (11). Additionally, miRNAs can typically inhibit the genes participated in the regulation of cellular processes including inflammation, cell cycle progression, stress response, cell differentiation and apoptosis (12). miRNAs control those cellular processes by regulation of oncogenes or anti-oncogenes, thereby affecting occurrence of gastric cancer (13). More importantly, it was documented that miRNAs play pivotal roles in mediation of gastric cancer stem cells and GC EMT (14, 15). For example, miR-375 functions as a tumor inhibitor to potentially modulate the growth of GC cells by targeting the oncogene JAK2 (16). A previous study revealed that miR-23a/b targets PDCD4 to induce tumorigenesis and to inhibit apoptosis of GC cells, suggesting an oncogenic role of miR-23a/b (17). Additionally, the downregulation of miR-23a has been shown to negatively regulate the tumor suppressor, phosphatase and tensin homologue (PTEN), to enhance the sensitivity of lung cancer stem cells to erotinib (18). Interestingly, the loss of PTEN facilitates cancer cell proliferation and contributes to tumorigenesis (19). In this regard, we speculated that exosomal miR-23a might target PTEN to contribute to gastric carcinogenesis. Based on previous researches, the current study was designed to investigate whether cancer cell-derived exosomes can carry miR-23a to promote the development of GC with the aim of identifying new therapeutic strategies as well as the underlying molecular mechanisms associated with PTEN.

## Materials and Methods

### Ethics Statement

The experiment was approved by the Ethics Committee of the First Affiliated Hospital of China Medical University and performed in strict accordance with the Declaration of Helsinki. All patients signed informed written consent documents.

### Microarray-Based Analysis

miRNA microarray data related to GC were retrieved from the Gene Expression Omnibus (GEO) database (https://www.ncbi.nlm.nih.gov/geo/), followed by differential analysis using the Limma package of R language. Next, a heatmap of the differentially expressed miRNAs was plotted using the pheatmap package. Expression of miR-23a in GC samples provided by the Cancer Genome Atlas (TCGA) was analyzed by starBase database (http://starbase.sysu.edu.cn/panCancer.php). The possible target genes of miR-23a were obtained using miRDB (http://starbase.sysu.edu.cn/panCancer.php), mirDIP (http://ophid.utoronto.ca/mirDIP/index.jsp#r), starBase (http://starbase.sysu.edu.cn/agoClipRNA.php?source=mRNA), and TargetScan (http://www.targetscan.org/vert_71/). MalaCards database (https://www.malacards.org/) was used to search the known GC-related genes, and subsequently STRING database (https://string-db.org/) was used to construct a gene interaction network map.

### Study Subjects

Forty GC patients who underwent GC resection at the First Affiliated Hospital of China Medical University from June 2018 to November 2018 were enrolled into the study. GC and adjacent normal tissues were collected from the participating patients. Among the enrolled patients, 22 cases were confirmed to be positive for hydrophobic-hydrophilic (HP^+^) and 18 were confirmed to be negative for HP (HP^−^). All tissue specimens were confirmed to be GC tissue by pathological examination, with none of the participants yet to receive local or systemic anti-tumor treatment prior to surgery. Patients who died from a non-GC or accident were excluded from the study. Following surgery, all tissue specimens were quickly frozen in liquid nitrogen for 1 h after being rinsed with normal saline, and stored at −80°C.

### Cell Treatment

The GC cell lines NCI-N87, HGC-27, AGS (Cell Bank of China Center for Type Culture Collection, Shanghai, China), MKN45 (CC-Y1358), and normal gastric mucosal epithelial cell line GES-1 (CC-Y1572) (EK-Bioscience, Shanghai, China) were subjected to mycoplasma test and short tandem repeat. The cells were cultured with Dulbecco's Modified Eagle's Medium (DMEM; Thermo Fisher Scientific Inc., Waltham, MA, USA) supplemented with 10% fetal bovine serum (FBS; Thermo Fisher Scientific Inc., Waltham, MA, USA.), 100 U/mL penicillin, and 100 μg/mL streptomycin.

The cells at logarithmic growth phase were seeded in a 6-well plate (6.0 × 10^5^ cells/well), and then transfected with negative control (NC)-mimic, miR-23a mimic, NC-inhibitor and miR-23a inhibitor as outlined in the instructions of Lipofectamine 2000 kit (Invitrogen, Carlsbad, CA, USA). After the transfected cells were cultured for 48 h, the exosomes were subsequently extracted.

### Extraction of Exosomes (20)

GC cell-derived exosomes were extracted by differential centrifugation. All centrifugation steps were performed at 4°C, and other steps were performed on ice. In short, cell debris and dead cells were removed following centrifugation at 500 g for 10 min and at 2,000 g for 20 min in a cryogenic centrifuge (Eppendorf, Germany). The supernatant was centrifuged at 110,000 g for 45 min in a high-speed centrifuge (Beckman, USA) to remove cell debris, large cells and extracellular vesicles. The supernatant was filtered using a 0.22 μm filter. Next, the exosomes were isolated at 110,000 g for 90 min in an ultracentrifuge (Beckman, USA). The collected pellets were resuspended in phosphate buffered saline (PBS) and repeatedly centrifuged once. Finally, the pellets were resuspended in 50–100 μL PBS and stored at −80°C for subsequent use. The exosome markers TSG101, CD63 and Alix were measured by Western blot analysis.

### Transmission Electron Microscope (TEM)

Firstly, the separated exosomes were thoroughly mixed with PBS. The sample was adsorbed on a carbon-coated nickel grid and counter-stained with 2% methyl tungstate for 5 min. The stain was then wiped off from the grid using tinder paper. After being dried, the samples were observed under a JEM-1230 electron microscope (Nihon Denshi, Tokyo, Japan) at an acceleration voltage of 80 kV.

### Nanoparticle Size Analysis

Firstly, the obtained exosome precipitate following centrifugation was suspended in 500 μL PBS at a ratio of 1:100 and stored at −20°C. Nanoparticle size measurement was then performed using a Nanosight LM10-HS nanoparticle analyzer (Great Malvern, UK).

### Reverse Transcription Quantitative Polymerase Chain Reaction (RT-qPCR)

The total RNA of the tissues or cells was extracted in strict accordance with the instructions of the TRIZOL kit (15596-018, Beijing solarbio science & technology Co., Ltd., Beijing, China), after which the concentration of RNA was determined. The obtained RNA was then subjected to reverse transcription using a miRNA reverse transcription kit (D1801, Harbin Xinhai Gene Co., Ltd., Harbin, China) and complementary DNA (cDNA) reverse transcription kit (K1622, Beijing Yaanda Biotechnology Co., Ltd., Beijing, China). Next, qPCR was performed on a ViiA 7 Real-Time PCR System (Daan Gene Co., Ltd., of Sun Yat-sen University, Guangzhou, China) with 2 μg total cDNA as template. U6 and GAPDH were used as internal references. The relative transcription levels of the target genes were calculated using the 2–^ΔΔCt^ method (21). The primers used in this study were synthesized by Takara (Dalian, China) (Table 1).

**Table 1:** Primer sequences for RT-qPCR.

**Gene**	**Primer sequence**
miR-23a	F: AGCAGCCAGTTACCCAAGA
	R: TGACAGTGCGAGACTCCATC
PTEN	F: GGACGAACTGGTGTAATGATATG
	R: TCTACTGTTTTTGTGAAGTACAGC
VEGF	F: CACATAGGAGAGATGAGCTTC
	R: CCGCCTCGGCTTGTCACAT
U6	F: CTCGCTTCGGCAGCACA
	R: AACGCTTCACGAATTTGCGT
GAPDH	F: GAAGGTGAAGGTCGGAGTCA
	R: AATGAAGGGGTCATTGATGG

*RT-qPCR, reverse transcription quantitative polymerase chain reaction; F, forward; R, reverse; miR-23a, microRNA-23a; PTEN, Phosphatase and tensin homologue deleted on chromosome 10; VEGF, vascular endothelial growth factor; GAPDH, glyceraldehyde-3-phosphate dehydrogenase*.

### Western Blot Analysis

Total protein was extracted by lysis for 15 min at 4°C and centrifugation at 15,000 × g for 15 min. Next, the supernatant was extracted, and the protein concentration was determined using a bicinchoninic acid kit (20201 ES76, Yeasen Biotechnology Co., Ltd., Shanghai, China). The protein was then separated by electrophoresis and then transferred onto a polyvinylidene fluoride membrane. The membrane was blocked with 5% bovine serum albumin for 1 h and incubated overnight at 4°C with the following primary antibodies: rabbit anti-human PTEN (1:10,000, ab32199, Abcam Inc., Cambridge, UK), mouse anti-human vascular endothelial growth factor (VEGF; 1:1,000, AV202-1, Beyotime Institute of Biotechnology, China), thrombospondin-1 (TSP-1; 1:10,000, ab85762, Abcam Inc., Cambridge, UK) and glyceraldehyde 3-phosphate dehydrogenase (GAPDH; 1:10,000, ab181602, Abcam Inc., Cambridge, UK). The membrane was then incubated with the horseradish peroxidase-labeled goat anti-rabbit immunoglobulin G (ab205718, 1:20,000, Abcam Inc., Cambridge, UK) for 1 h. The bands were developed using enhanced chemiluminescence and quantified using ImageJ 1.48u software (Wayne Rasband, National Institutes of Health, Bethesda, MD, USA). The ratio of the gray value of target proteins band to GAPDH band was expressed as the relative protein expression.

### Co-culture of Human Umbilical Venous Endothelial Cells (HUVECs) and GC Cells-Derived Exosomes

The exosomes were isolated from the HGC-27 cells transfected miR-23a inhibitor, NC-inhibitor, miR-23a mimic or NC-mimic (exo-miR-23a inhibitor, exo-NC-inhibitor, exo-miR-23a mimic, or exo-NC-mimic). The PBS-suspended exosomes were mixed with carboxy fluorescein succinimidyl ester (CFSE) (C1031, Beyotime Institute of Biotechnology, Shanghai, China) at a volume ratio of 10:1, and incubated at 37°C for 10 min, which was then terminated with 100 μL stop buffer followed by incubation at 4°C for 30 min. After 3-min centrifugation at 14,000 rpm, the fluorescence-labeled exosomes resuspended in 200 μL PBS were then mixed with exosome-free medium. The exosomes were co-cultured with the 50–60% confluent HUVECs. HUVECs were incubated only with PBS as control. Meanwhile, exo-miR-23a mimic was co-cultured with HUVECs transfected with overexpressed (oe)-PTEN or oe-NC. After incubation in 24-well plates for 48 h, the nuclei of the HUVECs were stained with 4',6-diamidino-2-phenylindole (DAPI, Abbott, USA) and subsequently observed under a confocal microscope (FV10i, Olympus Optical Co., Ltd, Tokyo, Japan) to determine the internalization of exosomes derived from the GC cells. Finally, the expression of miR-23a was quantified by RT-qPCR.

### 5-Ethynyl-2′-Deoxyuridine (EdU) Assay

The cells were seeded at a density of 5 × 10^4^ cells/mL. Based on the instructions of EdU kit (C10310, Ribobio, Guangzhou, China), the cells were incubated with EdU solution (100 μL/well) for 2 h, and fixed with cell fixative (100 μL/well) for 30 min at ambient temperature. The cells were incubated with 2 mg/mL glycine for 5 min, and permeabilized with PBS containing 0.5% TritonX-100 (100 μL/well) for 10 min. The cells were then incubated with 1 × Apollo reaction solution under conditions void of light for 30 min, and permeabilized with PBS containing 0.5% TritonX-100. The cells were then mounted with anti-fade mounting medium containing Hoechst. Image capture was performed under a fluorescence microscope, with the number of cells labeled with EdU recorded from three arbitrarily selected fields. The cells with nucleus stained in red were regarded as positive. EdU labeling rate (%) = the number of positive cells/(the number of positive cells + the number of negative cells) × 100%.

### *In vitro* Angiogenesis Assay

HUVECs (Catalog #5000, ScienCell Research Laboratories, Carlsbad, CA, USA) were cultured in endothelial cell growth medium (EGM-2) containing 10% FBS. Next, 20 μL BD Matrigel^TM^ Matrix was diluted using serum-free RPMI-1640 medium (total amount of 40 μL) at a ratio of 1:1, which was then added into the upper surface of polyester film in the Transwell chamber (membrane well size of 8 μm) and dried in a fume hood at room temperature for 1 h. Subsequently, 200 μL cell suspension (2 × 10^5^ cells/mL) was loaded to the apical chamber. The exosomes were then placed into the basolateral chamber. After 24 h of incubation in a 37°C wet incubator with 5% CO_2_, the branch nodes of the pseudo-tube-like and end-to-end tubular structure formed in HUVECs were observed in five randomly selected visual fields and numbered under an optical microscope.

### Dual Luciferase Reporter Gene Assay

The relationship between miR-23a and PTEN was predicted in the Targetscan website (http://www.targetscan.org), and further verified using dual luciferase reporter gene assay. The plasmids named PTEN-wild type (Wt) and PTEN-mutant (Mut) were constructed, after which the two plasmids were co-transfected into HEK-293T cells with miR-23a mimic and NC-mimic, respectively. After transfection for 24 h, the cells were lysed and centrifuged at 12,000 rpm for 1 min, followed by collection of the supernatant. Luciferase activity was then detected using the Dual-luciferase® Reporter Assay System (E1910, Promega, Madison, WI, USA).

### Statistical Analysis

All statistical analyses were conducted using SPSS 21.0 statistical software (IBM Corp. Armonk, NY, USA). Measurement data were expressed as mean ± standard deviation. If the data were in compliance with normal distribution and homogeneity of variance, paired data between two groups were compared using paired t test, and unpaired data between two groups were compared between using unpaired *t*-test. Comparisons among multiple groups were evaluated by one-way analysis of variance (ANOVA) with Tukey's *post-hoc* test. A value of *p* < 0.05 was considered to be indicative of statistical difference.

## Results

### High Expression of miR-23a Is Detected in GC Tissues and Cells

**Figure 1:**
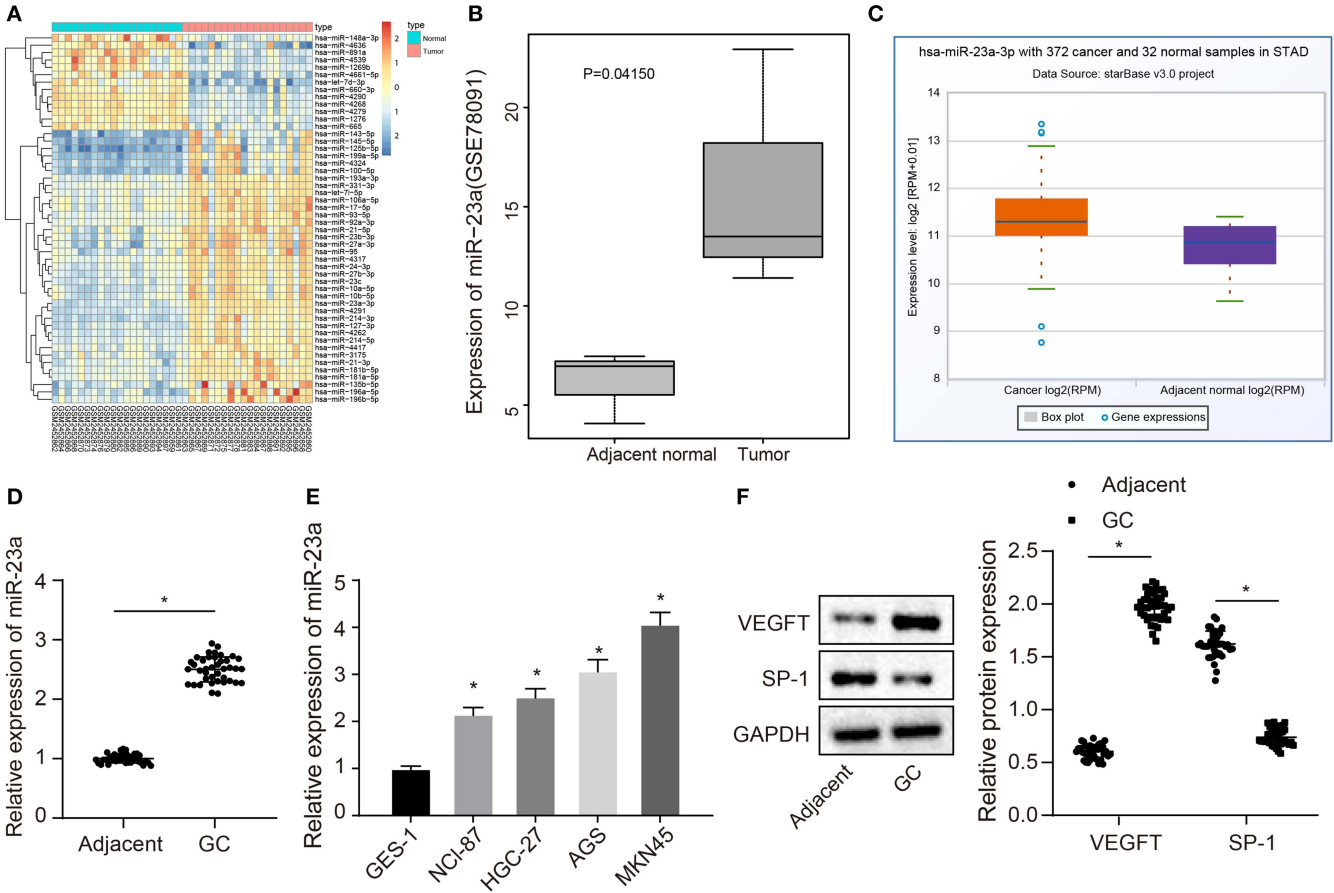
miR-23a is expressed highly in GC tissues and cells. **(A)** Differentially expressed miRNAs in the dataset GSE93415, the abscissa represents the sample number, the ordinate represents the names of miRNA, and the left dendrogram indicates the miRNA expression cluster. Each rectangle represents the expression of a miRNA in a sample. **(B)** Expression of miR-23a in the dataset GSE78091, the abscissa refers to the sample type, the ordinate refers to the expression of miRNA, the left box plot refers to the normal sample, and the right box plot refers to the tumor sample. **(C)** The expression of miR-23a in the tumor sample (left box) and normal sample (right box) in the TCGA database. **(D)** Determination of miR-23a expression in GC tissues and adjacent normal tissues by RT-qPCR. **(E)** Determination of miR-23a expression in human gastric normal mucosal epithelial cell line and GC cell lines by RT-qPCR. **(F)** Western blot analysis of VEGF and TSP-1 proteins in GC tissues and adjacent normal tissues. Measurement data were expressed as mean ± standard deviation. Data between two groups were compared using paired *t*-test (*n* = 40). Comparisons among multiple groups were conducted by one-way ANOVA with Tukey's *post hoc* test. The experiment was repeated three times independently. *p < 0.05, there was statistical difference.

The GC-associated miRNA expression dataset GSE93415 was obtained through retrieval from the GEO database. The results indicated 76 differently expressed miRNAs in GC. The heatmap depicted the top 50 miRNAs exhibiting larger fold changes (Figure 1A). Among them, miR-23a was the miRNA with the largest fold change. Meanwhile, based on the GSE78091 dataset retrieved from the GEO database and data from TCGA, we detected that miR-23a was robustly induced in GC (Figures 1B,C). High expression of miR-23a in GC was further demonstrated by RT-qPCR. As illustrated in Figure 1D, compared with adjacent normal tissues, miR-23a expression was appreciably increased in GC tissues. Further correlation analysis exhibited that miR-23a expression was positively with tumor node metastasis (TNM) and not correlated with age, histological grade, and gender (Table 2). Subsequently, RT-qPCR data displayed remarkably higher miR-23a expression in GC cell lines NCI-N87, HGC-27, AGS, and MKN45 than in normal mucosal epithelial cell line GES-1 (Figure 1E). These results confirmed that miR-23a was highly expressed in GC tissues and cells. In addition, Western blot analysis exhibited that compared with adjacent normal tissues, the protein expression of VEGF was notably increased, but that of TSP-1 was diminished in GC tissues (Figure 1F), indicating that tube formation was more prominent in GC tissues.

**Table 2:** Correlation between miR-23a expression and clinicopathological characteristics.

	**Variables**	**Expression**		**Total**	**χ** **^2^**	***p*-value**
		**Low**	**High**			
Age (year)					0.44	0.507
	≤60	12	14	26		
	>60	8	6	14		
Histological grade					2.667	0.103
	Low	10	15	25		
	High	10	5	15		
Sex					2.506	0.113
	Female	7	12	19		
	Male	13	8	21		
TNM stage					17.29	<0.001
	I/II	18	5	23		
	III/IV	2	15	17		
N stage					10.16	0.001
	N0	19	10	29		
	N1/N2/N3	1	10	11		
M stage					8.64	0.003
	M0	17	8	25		
	M1	3	12	15		

### miR-23a Is Enriched in GC Cells-Derived Exosomes

**Figure 2:**
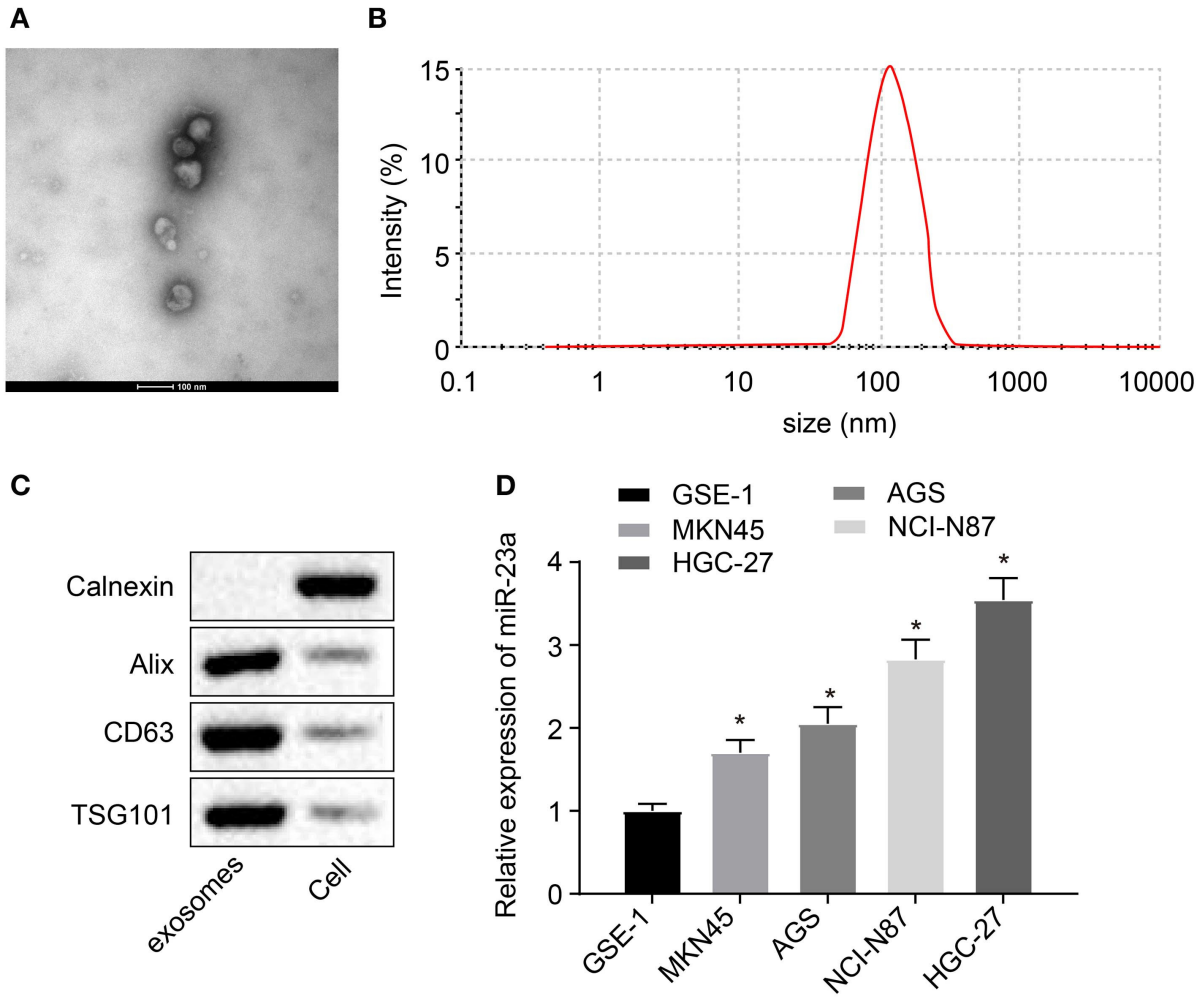
miR-23a exists in the exosomes derived from GC cells. **(A)** Observation of exosome morphology by transmission electron microscope (scale bar = 100 nm), red arrows refer to exosome. **(B)** Nanoparticle size analysis of exosomes. **(C)** Western blot analysis of TSG101, CD63 and Alix proteins in isolated exosomes. **(D)** Determination of miR-23a expression in exosomes isolated from normal gastric cell lines and GC cell lines by RT-qPCR. *p < 0.05 vs. GSE-1 cell line. Measurement data were expressed as mean ± standard deviation. Comparisons among multiple groups were conducted by one-way ANOVA with Tukey's *post hoc* test. The experiment was repeated three times independently.

Studies have shown that miR-23a can be carried by tumor cell-secreted exosomes such as nasopharyngeal carcinoma or lung cancer cells and then exert their regulatory functions on angiogenesis (22–24), which suggested that miR-23a carried by GC cell-derived exosomes might be involved in angiogenesis. To validate this hypothesis, exosomes were extracted from GES-1, NCI-N87, HGC-27, AGS, and MKN45 using the ExoQuick method and then identified. The ultrastructure of exosomes was observed under the TEM. It can be clearly observed that the extracts exhibited typical morphological features of exosomes, varying in diameter, with a spherical structure formed by a lipid bilayer molecular membrane. The outer layer of two-layer lipid molecular membrane was deeply dyed, and the interior was heterogeneously light-stained, where protein density substance was visible (Figure 2A). Subsequent results of nanoparticle size analysis (Figure 2B) displayed that the diameter of extracted exosomes was around 100 nm. Next, expression of exosome surface markers (CD63, Alix, and TSG101) in GC cells was highly expressed in exosomes (Figure 2C). RT-qPCR was used to measure the expression of miR-23a in exosomes derived from normal gastric cell line GES-1 and GC cell lines. As depicted in Figure 2D, compared with GES-1 cell-derived exosomes, miR-23a expression in GC cell-derived exosomes was significantly increased, and HGC-27 cell-derived exosomes exhibited the highest expression of miR-23a. Therefore, HGC-27 cells were chosen for the subsequent experiments. The aforementioned results demonstrated the enrichment of miR-23a in the exosome derived from GC cells.

### Co-culture of HUVECs and GC Cell-Derived Exosomes Stimulates Angiogenesis

**Figure 3:**
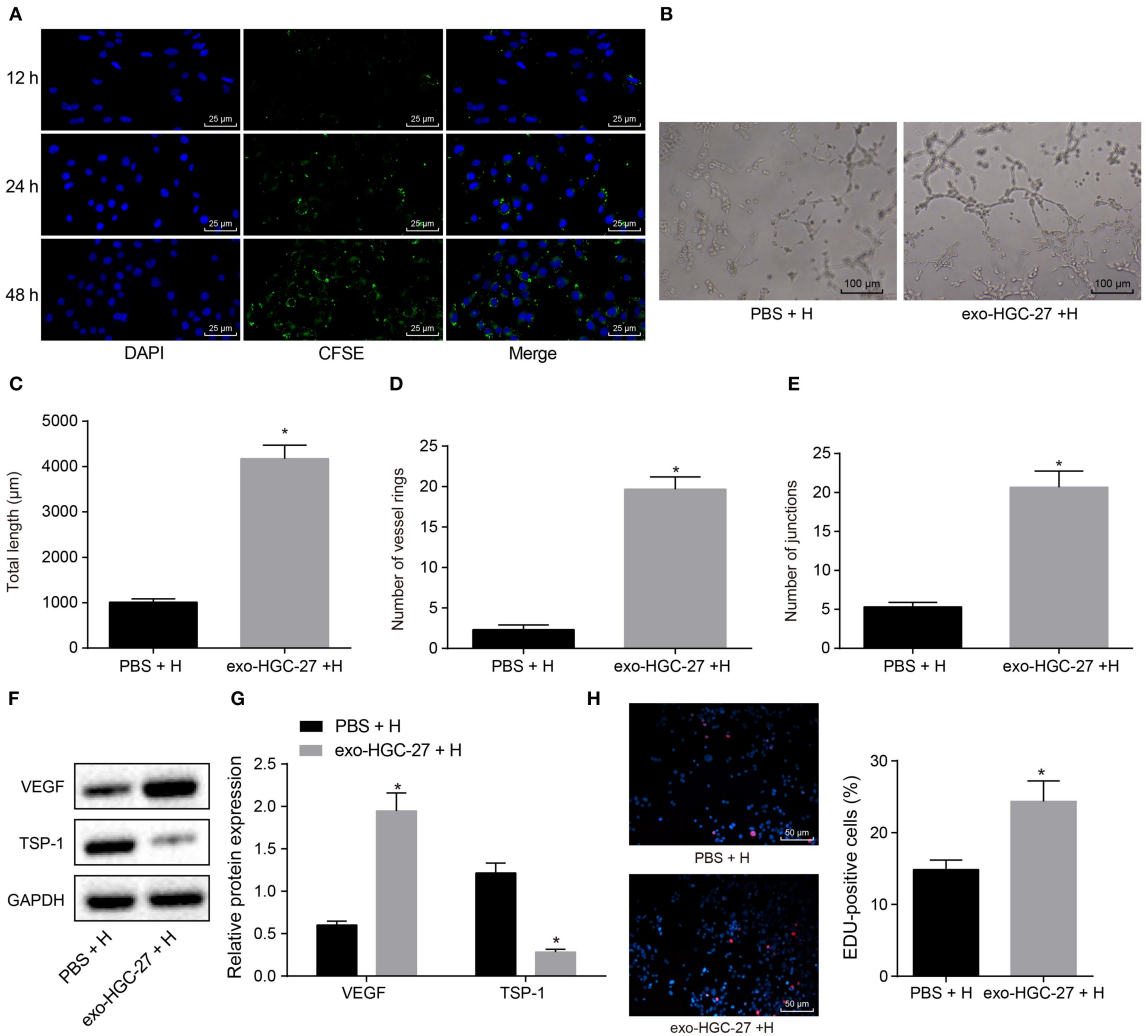
Exosomes derived from GC cells enhances angiogenesis of HUVECs in a co-culture system. **(A)** Internalization of exosomes by HUVECs observed under laser confocal microscope (×400). **(B–E)** Tube formation (×100), tube length, loop number and tube nodes in HUVECs after co-culture with exosomes or PBS. **(F,G)** Western blot analysis of VEGF and TSP-1 proteins. **(H)** The proliferation of HUVECs assessed by EdU labeling (×200). *p < 0.05 vs. HUVECs co-cultured with PBS. The data in the figure were measurement data, which were expressed as mean ± standard deviation. If the data were in compliance with normal distribution and homogeneity, comparisons between two groups were conducted using unpaired *t*-test. The experiment was repeated three times independently.

In order to clarify whether co-culture of HUVECs and GC cell-derived exosomes could promote angiogenesis, HUVECs were co-cultured with HGC-27-derived exosomes or PBS. CFSE was used to label exosomes (green) and DAPI to label nuclei of HUVECs (blue), and then the exosome internalization of HUVECs was observed under the confocal microscope. More CFSE-exosomes were internalized by HUVECs with the time prolonged (Figure 3A). As depicted in Figures 3B–E, tube formation ability of HUVECs co-cultured with HGC-27-derived exosomes was enhanced, evidenced by increased tube length and number of loops and nodes. In addition, expression of VEGF and TSP-1 upon co-culture of HUVECs with HGC-27-derived exosomes was measured by Western blot analysis. The results (Figures 3F,G) presented that the protein expression of VEGF upon co-culture of HUVECs with HGC-27-derived exosomes was notably increased, while that of TSP-1 was reduced, which further confirmed that exosomes from GC cells can facilitate the tube formation ability. EdU assay consistently documented (Figure 3H) that the proliferation of HUVECs co-cultured with HGC-27-derived exosomes was markedly increased. Based on the above results, it can be concluded that exosomes derived from GC cells can promote angiogenesis and proliferation of HUVECs.

### Transfer of miR-23a via GC Cell-Derived Exosomes Promotes Angiogenesis

**Figure 4:**
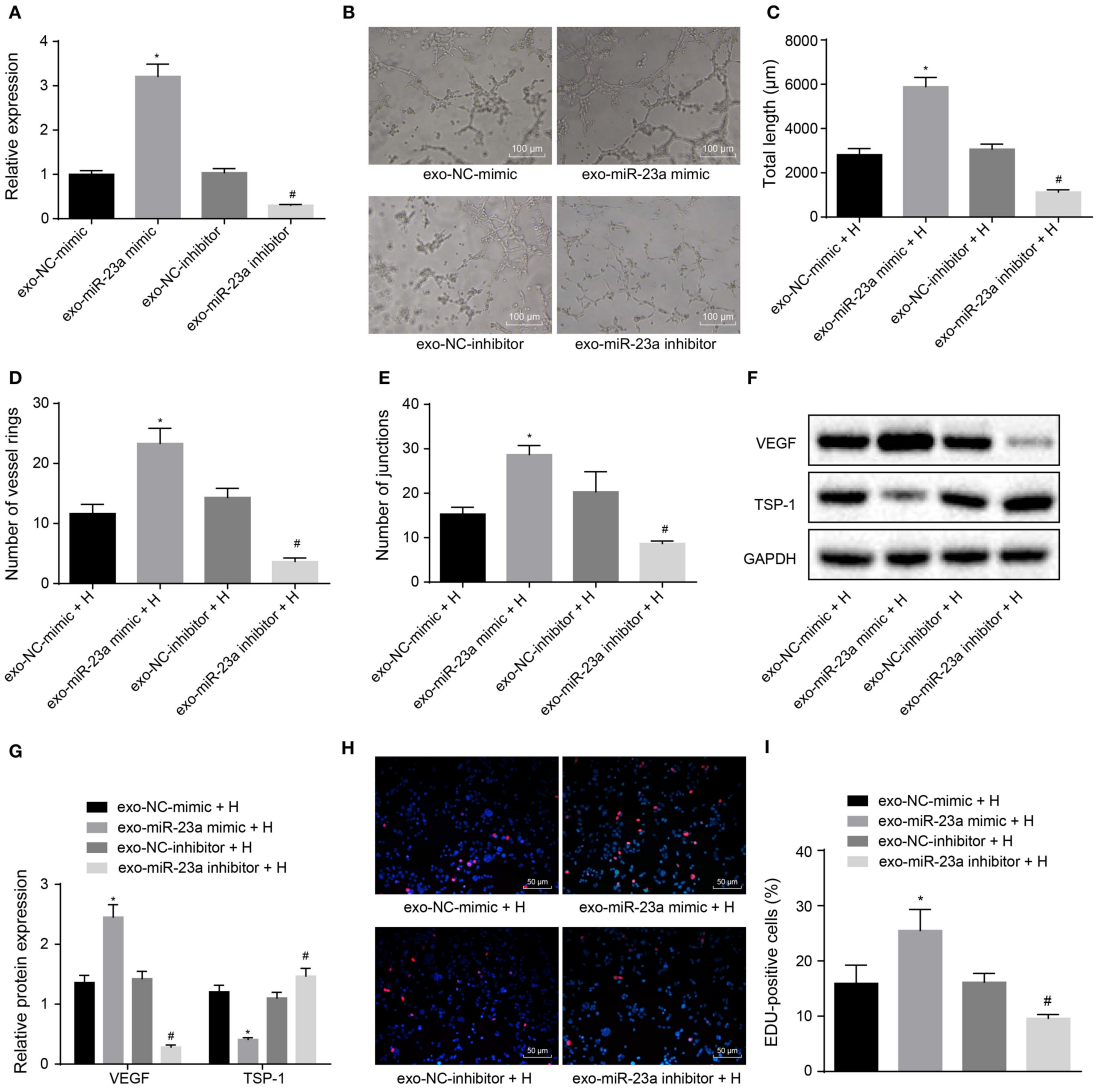
GC cells-derived exosomal miR-23a promotes *in vitro* angiogenesis. HGC-27 cells were transfected with miR-23a mimic or inhibitor and their derived exosomes were co-cultured with HUVECs. **(A)** RT-qPCR determination of miR-23a expression. **(B)** Representative images of the tube formation in HUVECs (×100). **(C–E)** The tube length, number of loops and nodes of HUVECs. **(F,G)** Western blot analysis of VEGF and TSP-1 proteins. **(H,I)** Representative images of EdU-positive cells and proliferation rate of HUVECs (×200). *p < 0.05 vs. HUVECs co-cultured with exosomes derived from NC-mimic-transfected HGC-27 cells. ^#^*p* < 0.05 vs. HUVECs co-cultured with exosomes derived from NC inhibitor-transfected HGC-27 cells. Measurement data were expressed as mean ± standard deviation. If the data were in compliance with normal distribution and homogeneity, comparisons between two groups were conducted using unpaired *t*-test. The experiment was repeated three times independently.

To further elucidate the effects of GC cell-derived exosomes on tube formation via miR-23a, HGC-27 cells were transfected with miR-23a mimic or inhibitor to alter endogenous miR-23a level. RT-qPCR revealed that miR-23a expression was upregulated in exosomes derived from HGC-27 cells that had been transfected with miR-23a mimic, whereas that was decreased in exosomes derived from miR-23a inhibitor-transfected HGC-27 cells (Figure 4A). As illustrated in Figures 4B–E, the tube formation ability of HUVECs co-cultured with exosomes derived from miR-23a mimic-transfected HGC-27 cells was enhanced, and the tube length and number of loops and nodes were potently increased. Conversely, the tube formation ability of HUVECs co-cultured with exo-miR-23a inhibitor was inhibited. Western blot analysis subsequently manifested that the protein expression of VEGF was strikingly elevated while that of TSP-1 was diminished in HUVECs co-cultured with exosomes derived from miR-23a mimic-transfected HGC-27 cells (Figures 4F,G). On the contrary, the protein expression of VEGF was significantly reduced while that of TSP-1 was elevated in HUVECs co-cultured with exosomes derived from miR-23a inhibitor-transfected HGC-27 cells. The EdU assay results (Figures 4H,I) displayed that the proliferation of HUVECs co-cultured with exosomes derived from miR-23a mimic-transfected HGC-27 cells was significantly increased, but that of HUVECs co-cultured with exosomes derived from miR-23a inhibitor-transfected HGC-27 cells was obviously reduced. Taken together, exosomal miR-23a secreted from GC cells can promote tube formation.

### PTEN Is a Target Gene of miR-23a

**Figure 5:**
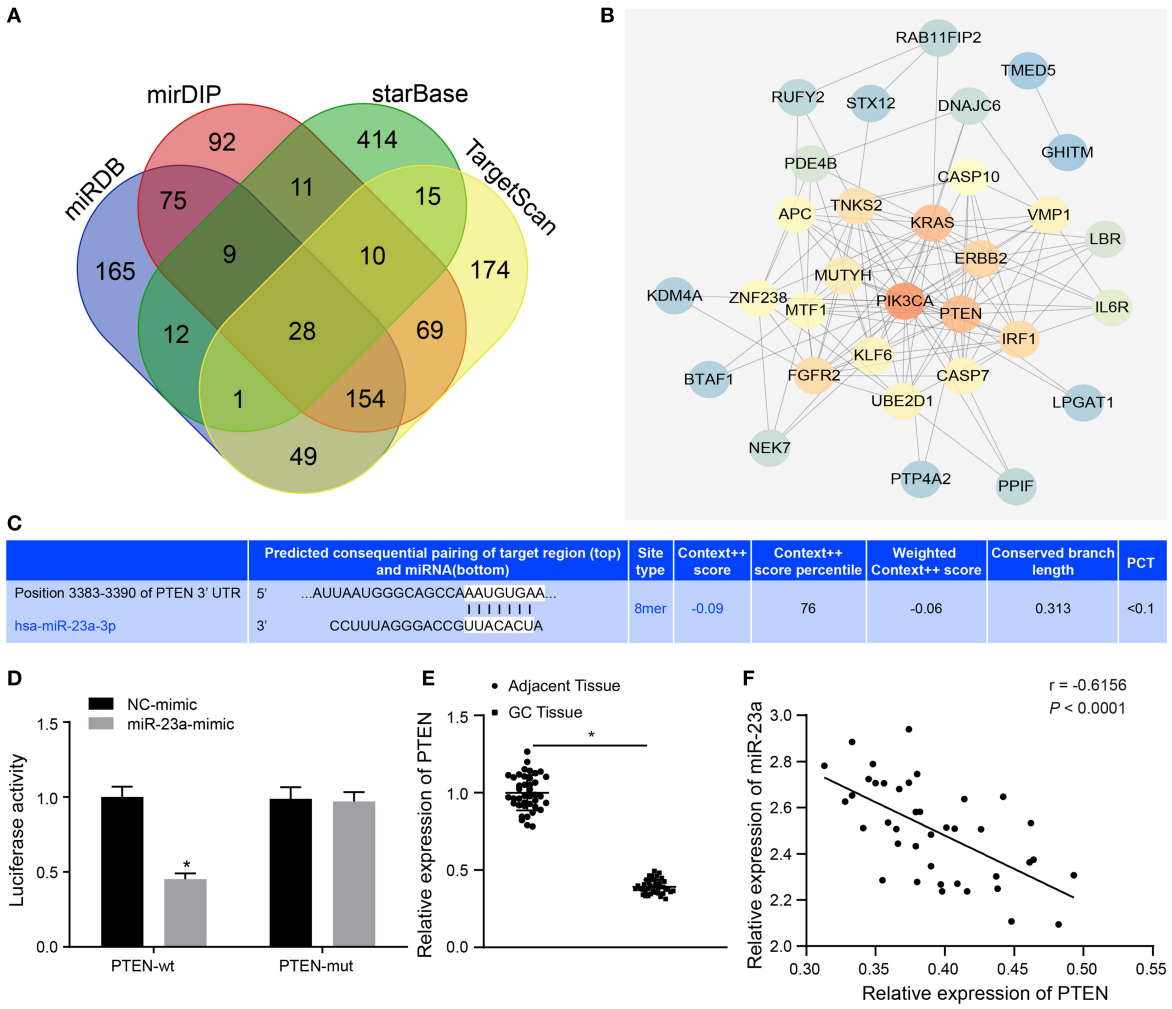
miR-23a targets PTEN and negatively regulated its expression. **(A)** Intersection of predicted target genes of miR-23a based on the results of four databases, where the middle part represents the intersection. **(B)** Correlation analysis of the known genes related to GC with target genes of miR-23a. Each small circle in the figure represents a gene, and the line between the circles indicates the interaction between two genes. **(C)** The predicted binding sites of miR-23a on the PTEN by TargetScan website. **(D)** The binding relationship between miR-23a and PTEN confirmed by dual-luciferase reporter gene assay. **(E)** PTEN expression in GC tissues and adjacent normal tissues detected by RT-qPCR. **(F)** Correlation between miR-23a and PTEN expression analyzed by Pearson. *p < 0.05 vs. cells transfected with NC-mimic. Measurement data were expressed as mean ± standard deviation. If the data were in compliance with normal distribution and homogeneity, comparisons between two groups were conducted using unpaired *t*-test. The experiment was repeated three times independently.

To further understand the mechanism of miR-23a in GC, miRDB, mirDIP, starBase and TargetScan databases were used to predict the downstream genes of miR-23a. To improve the prediction accuracy, the target genes with the scores higher than 70 points predicted by miRDB and those with the scores higher than 0.7 points in mirDIP along with the top 500 target genes in the remaining two databases were intersected, and 28 overlapping potential target genes were finally obtained (Figure 5A). Simultaneously, the MalaCards database was adopted to retrieve the most likely genes involved in GC development from these 28 potential target genes, which predicted 10 known genes related to GC. Next, correlation analysis was conducted for the GC-related known genes and the potential target genes of miR-23a. According to the gene interaction network map, the PTEN gene was located at the core (Figure 5B). Next, TargetScan database (http://www.targetscan.org) predicted a binding site between miR-23a and PTEN (Figure 5C). In order to confirm their relationship, dual-luciferase reporter gene assay was performed. As depicted in Figure 5D, the luciferase activity of PTEN-3′-UTR-Wt was significantly inhibited by miR-23a mimic, while that of PTEN-3′-UTR-Mut remained unaffected. Moreover, RT-qPCR revealed that PTEN expression was remarkably lower in GC tissues than in adjacent normal tissues (Figure 5E). Through correlation analysis, it was observed that there was inverse correlation between miR-23a and PTEN expression (Figure 5F). Further, PTEN expression was negatively with TNM, and had no significant relationship to age, histological grade and gender (Table 3). Therefore, miR-23a directly targeted PTEN.

**Table 3:** Correlation between miR-23a expression and clinicopathological characteristics.

	**Variables**	**Expression**		**Total**	**χ** **^2^**	***p*-value**
		**Low**	**High**			
Age (year)					1.192	0.275
	≤60	11	15	26		
	>60	9	6	14		
Histological grade					0.007	0.936
	Low	14	11	25		
	High	6	5	15		
Sex					0.742	0.342
	Female	11	8	19		
	Male	9	12	21		
TNM stage					12.38	0.0004
	I/II	6	17	23		
	III/IV	14	3	17		
N stage					6.144	0.013
	N0	11	18	29		
	N1/N2/N3	12	8	11		
M stage					5.227	0.022
	M0	9	16	25		
	M1	11	4	15		

### Exosomal miR-23a Promotes Angiogenesis and Activates the AKT Pathway by Inhibiting PTEN

**Figure 6:**
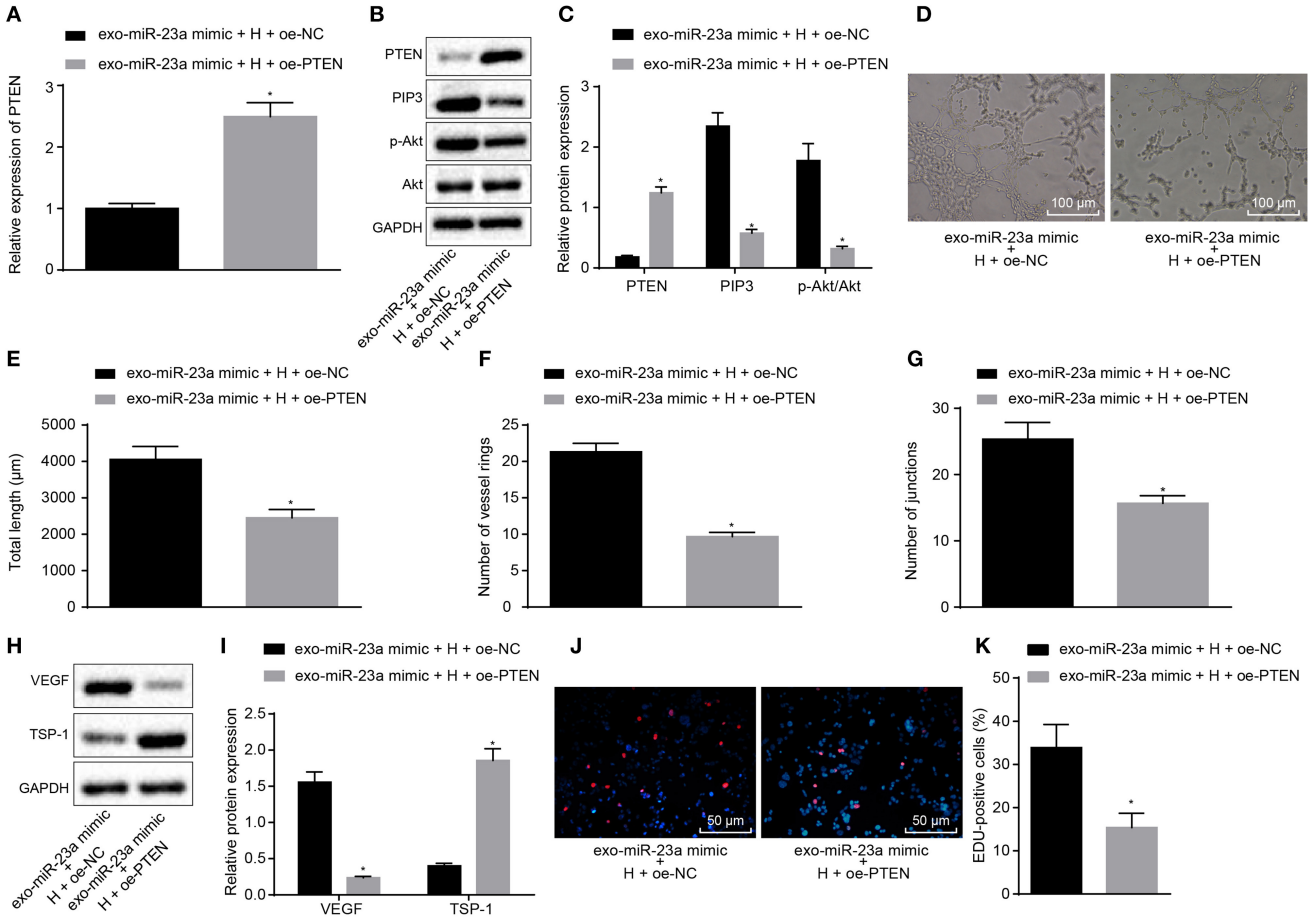
GC cell-derived exosomal miR-23a accelerates angiogenesis through inhibition of PTEN expression. **(A)** Determination of PTEN mRNA expression by RT-qPCR. **(B,C)** Western blot analysis of PTEN, PIP3, phosphorylated Akt and Akt proteins. **(D)** Representative images of the tube formation in HUVECs (×100). **(E–G)** The tube length, number of loops and nodes in HUVECs. **(H,I)** Western blot analysis of VEGF and TSP-1 proteins in HUVECs. **(J,K)** The proliferation of HUVECs assessed by EdU assay (×200). *p < 0.05 vs. HUVECs co-cultured with exosomes derived from miR-23a-mimic and oe-NC-transfected HGC-27 cells. Measurement data were expressed as mean ± standard deviation. If the data were in compliance with normal distribution and homogeneity, comparisons between two groups were conducted using unpaired *t*-test. The experiment was repeated three times independently.

To verify that miR-23a inhibits PTEN expression to promote angiogenesis, oe-PTEN-transfected HUVECs were co-cultured with the exosomes derived from miR-23a mimic-transfected HGC-27 cells. RT-qPCR (Figure 6A) and Western blot analysis (Figures 6B,C) provided data showing that mRNA and protein expression of TSP-1 was increased substantially in oe-PTEN-transfected HUVECs co-cultured with exosomes-miR-23a mimic, but that of PIP3 and phosphorylated Akt/Akt was reduced as compared to the oe-NC transfected HUVECs co-cultured with exosomes-miR-23a mimic. Subsequent results from tube formation experiment (Figures 6D–G) showed that the ability of tube formation was weakened in oe-PTEN-transfected HUVECs co-cultured with exosomes-miR-23a mimic, which was indicated by diminished tube length and number of loops and nodes, in contrast to oe-NC transfected HUVECs co-cultured with exosomes-miR-23a mimic. At the same time, Western blot analysis (Figures 6H,I) showed that the protein expression of TSP-1 was significantly increased in oe-PTEN-transfected HUVECs co-cultured with exosomes-miR-23a mimic, but that of VEGF was significantly reduced in contrast to oe-NC transfected HUVECs co-cultured with exosomes-miR-23a mimic. EdU assay results suggested that the proliferation of oe-PTEN-transfected HUVECs co-cultured with exosomes-miR-23a mimic was significantly reduced vs. that of oe-NC transfected HUVECs co-cultured with exosomes-miR-23a mimic (Figures 6J,K). Altogether, exosomal miR-23a could promote angiogenesis via inhibition of PTEN.

## Discussion

Significant implications regarding GC cell-derived miRNAs in the processes of tumorigenesis and metastasis have been previously emphasized in literature (25). miRNAs function as oncomiRs or tumor suppressive miRs in the carcinogenesis and development of upper gastrointestinal tract cancers, including GC (26). The current study placed particular emphasis on the functions of exosomal miR-23a in GC, and subsequently obtained data confirming that exosomal miR-23a induced GC progression by promoting angiogenesis via PTEN.

A key initial finding of our study revealed that miR-23a was expressed at a high level in GC tissues and cells. Current studies have widely explored the role of miR-23a in human cancers. For example, miR-23a accelerates autophagy and increases survival and migration of breast cancer cells by targeting XIAP (27). A previous study concluded that miR-23a up-regulates IKK alpha expression while down-regulating the expression of ST7L expression, ultimately enhancing the malignant phenotypes of epithelial ovarian cancer cells (28). The apoptosis of GC cells can be inhibited by miR-23a by down-regulating PPP2R5E (29). Additionally, miR-23a targets metallothionein 2A and consequently facilitates the growth of GC cells (30). Moreover, miR-23a has been reported to promote GC cell proliferation and metastatic potential by targeting SPRY2 (31). Hence, gain-of-function of miR-23a exhibits a promotive effect on tumor progression, which may function by targeting some tumor suppressors.

In the present study, PTEN was found to be under-expressed in GC tissues, which was further verified as a target of miR-23a. PTEN is a recognized tumor suppressor in various kinds of human cancers including breast cancer (32). High expression of PTEN is conducive to restrain cancer cell proliferation and decrease cell survival in colorectal cancer (33). It is reported that deletion of PTEN contributes to the occurrence and development of GC (34). PTEN can be targeted by miR-718 and miR-382 and is consistently shown to inhibit the angiogenesis and progression of GC (35). Thus, miR-23a potentially played a promoting role in GC angiogenesis and progression by negatively regulating PTEN.

We also validated that miR-23a was enriched in cancer-derived exosomes, and exosomes-delivered miR-23a facilitated angiogenesis by targeting PTEN in a co-culture system. Partially, consistent with our findings, exosomes secreted by hypoxic lung cancer cells carry miR-23a to promote angiogenesis and capillary permeability (24). Similarly, lung cancer cells-derived extracellular vesicles have been shown to enhance endothelial cell angiogenesis and radiation resistance by transferring miR-23a which functionally down-regulates PTEN (36). However, this study further identified an involvement of the Akt pathway during the regulation process of miR-23a in tumor angiogenesis. The phosphatidylinositol 3-kinase (PI3K)/Akt pathway is over-activated in GC (37), and modulates a wide range of cellular processes, such as proliferation and angiogenesis (38). Additionally, restoration of PTEN expression may block angiogenesis in GC by inactivating the PI3K/Akt pathway (39). The loss of PTEN has been reported to trigger activation of the PI3K/Akt pathway, which enhances angiogenesis (40). Based on the aforementioned references and our results, it can be inferred that exosomal miR-23a may promote angiogenesis by activating the PTEN-dependent Akt pathway, which may stimulate the deterioration of cancer cells.

This study also verified the angiogenic role of exosomes-delivered miR-23a in molecular levels, as our data shown that VEGF expression was markedly increased, while TSP-1 expression was reduced by GC cell-derived exosomal miR-23a. VEGF, one of the powerful activators of angiogenesis, stimulates endothelial cell migration and proliferation abilities in existing vessels, thereby enhancing the generation and stabilization of new blood vessels (41, 42). TSP-1 is a multidomain glycoprotein participated in intercellular and cell to matrix interactions, and particularly related to angiogenesis and tumorigenesis (43). Furthermore, overexpression of TSP-1 is correlated with suppression of angiogenesis in human cancers such as prostate cancer (44). Those findings contribute to the conception proposed by our study that GC cell-derived exosomal miR-23a facilitates angiogenesis, as shown by increased VEGF but reduced TSP-1 levels.

Taken together, this study corroborated that miR-23a carried by exosomes inhibited PTEN to promote the development of GC by inducing angiogenesis. Based on the above findings, miR-23a may be a promising target for GC treatment. Altogether, this study may open new avenues of research to harness the role of miR-23a in optimizing future endothelial cell-based therapies for GC. However, there were several limitations in our study. Firstly, the sample size in our study was relatively small, so further studies with larger sample sized should be conducted in the future. Secondly, due to the limited funding and technology, *in vivo* assays cannot be carried out temporarily. Thirdly, we only used a single dose of exosomes in our experiments. It will be necessary to perform extended time course and dose-response experiments in follow-up studies examining the translational potential of these findings. Finally, investigation into efficient, economical and effective exosome isolation techniques requires future exploration.

## Data Availability Statement

The datasets generated for this study are available on request to the corresponding author.

## Ethics Statement

The studies involving human participants were reviewed and approved by the First Affiliated Hospital of China Medical University. The patients/participants provided their written informed consent to participate in this study.

## Author Contributions

JD, YL, JL, JMZ, and XYL designed the study. JD and YL collated the data, carried out data analyses, and produced the initial draft of the manuscript. JL, JMZ, and XYL contributed to drafting the manuscript. All authors have read and approved the final submitted manuscript.

### Conflict of Interest

The authors declare that the research was conducted in the absence of any commercial or financial relationships that could be construed as a potential conflict of interest.
