# Corrigendum: Gastric Cancer Cell-Derived Exosomal MicroRNA-23a Promotes Angiogenesis by Targeting PTEN

**DOI:** 10.3389/fonc.2021.797657

**Published:** 2022-01-20

**Authors:** Jiang Du, Yuan Liang, Ji Li, Jin-Ming Zhao, Xu-Yong Lin

**Affiliations:** ^1^ Department of Pathology, The First Affiliated Hospital and College of Basic Medical Science, China Medical University, Shenyang, China; ^2^ Medical Oncology Department of Thoracic Cancer (2), Cancer Hospital of China Medical University, Liaoning Cancer Hospital & Institute, Shenyang, China

**Keywords:** exosome, gastric cancer, microRNA-23a, PTEN, AKT pathway, angiogenesis

Zhen-Ning Wang was included as an author in the published article and he should be removed from the authorship. The corrected Author Contributions Statement appears below. The authors apologize for this error and state that this does not change the scientific conclusions of the article in any way. The original article has been updated.

JD, YL, JL, JMZ, and XYL designed the study. JD and YL collated the data, carried out data analyses, and produced the initial draft of the manuscript. JL, JMZ, and XYL contributed to drafting the manuscript. All authors have read and approved the final submitted manuscript.

In the original article, there was an error. The cell line sources listed in the “Cell Treatment” section were incorrect due to a translation error.

A correction has been made to **Materials and Methods**, *“Cell Treatment”*, paragraph 1:

“The GC cell lines NCI-N87, HGC-27, AGS (Cell Bank of China Center for Type Culture Collection, Shanghai, China), MKN45 (CC-Y1358), and normal gastric mucosal epithelial cell line GES-1 (CC-Y1572) (EK-Bioscience, Shanghai, China) were subjected to mycoplasma test and short tandem repeat. The cells were cultured with Dulbecco’s Modified Eagle’s Medium (DMEM; Thermo Fisher Scientific Inc., Waltham, MA, USA) supplemented with 10% fetal bovine serum (FBS; Thermo Fisher Scientific Inc., Waltham, MA, USA.), 100 U/mL penicillin, and 100 μg/mL streptomycin.”

In the original article, there was a mistake in **Figures 5** and **6** as published. Modifications to the targeted verification in **Figure 5** and Akt protein typographical errors in the WB experiment in **Figure 6** were made after a recheck of the figures, but not included in the final article. The corrected **Figures 5** and **6** appear below.

**Figure 5:**
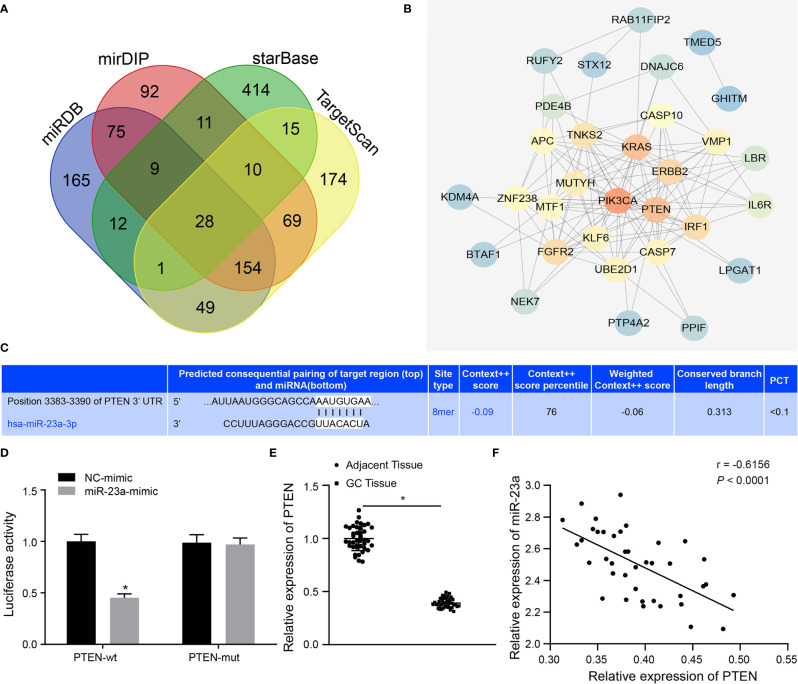
miR-23a targets PTEN and negatively regulated its expression. **(A)** Intersection of predicted target genes of miR-23a based on the results of four databases, where the middle part represents the intersection. **(B)** Correlation analysis of the known genes related to GC with target genes of miR-23a. Each small circle in the figure represents a gene, and the line between the circles indicates the interaction between two genes. **(C)** The predicted binding sites of miR-23a on the PTEN by TargetScan website. **(D)** The binding relationship between miR-23a and PTEN confirmed by dual-luciferase reporter gene assay. **(E)** PTEN expression in GC tissues and adjacent normal tissues detected by RT-qPCR. **(F)** Correlation between miR-23a and PTEN expression analyzed by Pearson. *p < 0.05 vs. cells transfected with NC-mimic. Measurement data were expressed as mean ± standard deviation. If the data were in compliance with normal distribution and homogeneity, comparisons between two groups were conducted using unpaired t-test. The experiment was repeated three times independently.

**Figure 6:**
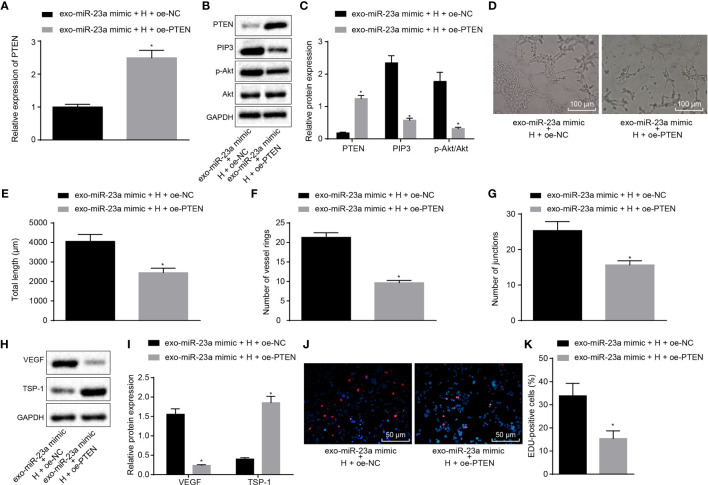
GC cell-derived exosomal miR-23a accelerates angiogenesis through inhibition of PTEN expression. **(A)** Determination of PTEN mRNA expression by RT-qPCR. **(B, C)** Western blot analysis of PTEN, PIP3, phosphorylated Akt and Akt proteins. **(D)** Representative images of the tube formation in HUVECs (×100). **(E–G)** The tube length, number of loops and nodes in HUVECs. (H,I) Western blot analysis of VEGF and TSP-1 proteins in HUVECs. **(J, K)** The proliferation of HUVECs assessed by EdU assay (×200). *p < 0.05 vs. HUVECs co-cultured with exosomes derived from miR-23a-mimic and oe-NC-transfected HGC-27 cells. Measurement data were expressed as mean ± standard deviation. If the data were in compliance with normal distribution and homogeneity, comparisons between two groups were conducted using unpaired t-test. The experiment was repeated three times independently.

The authors apologize for these errors and state that this does not change the scientific conclusions of the article in any way. The original article has been updated.

## Publisher’s Note

All claims expressed in this article are solely those of the authors and do not necessarily represent those of their affiliated organizations, or those of the publisher, the editors and the reviewers. Any product that may be evaluated in this article, or claim that may be made by its manufacturer, is not guaranteed or endorsed by the publisher.

